# Cell-free DNA methylation analysis as a marker of malignancy in pleural fluid

**DOI:** 10.1038/s41598-024-53132-x

**Published:** 2024-02-05

**Authors:** Billie Bixby, Lukas Vrba, Jyoti Lenka, Marc M. Oshiro, George S. Watts, Trina. Hughes, Heidi Erickson, Madhav Chopra, James L. Knepler, Kenneth S. Knox, Lisa Jarnagin, Raed Alalawi, Mrinalini Kala, Richard Bernert, Joshua Routh, Denise J. Roe, Linda L. Garland, Bernard W. Futscher, Mark A. Nelson

**Affiliations:** 1 Pulmonary, Critical Care, Allergy and Sleep Medicine, Department of Medicine, University of Arizona, Tucson, USA; 2 Precision Epigenomics, Tucson, AZ USA; 3 Boyer Liver Institute, Department of Medicine, University of Arizona, Tucson, USA; 4 Department of Pharmacology and Toxicology, University of Arizona, Tucson, USA; 5 Pulmonary, Critical Care, Allergy and Sleep Medicine, Department of Medicine, University of Arizona, Phoenix, USA; 6 Department of Internal Medicine, University of Arizona, Phoenix, USA; 7 Midwestern University, Glendale, USA; 8 Mel and Enid Zuckerman College of Public Health, University of Arizona, Tucson, USA; 9 Hematology Oncology, Department of Medicine, University of Arizona, Tucson, USA; 10 Department of Pathology, University of Arizona, Tucson, AZ 85724 USA

**Keywords:** Cancer, Tumour biomarkers

## Abstract

Diagnosis of malignant pleural effusion (MPE) is made by cytological examination of pleural fluid or histological examination of pleural tissue from biopsy. Unfortunately, detection of malignancy using cytology has an overall sensitivity of 50%, and is dependent upon tumor load, volume of fluid assessed, and cytopathologist experience. The diagnostic yield of pleural fluid cytology is also compromised by low abundance of tumor cells or when morphology is obscured by inflammation or reactive mesothelial cells. A reliable molecular marker that may complement fluid cytology for the diagnosis of malignant pleural effusion is needed. The purpose of this study was to establish a molecular diagnostic approach based on pleural effusion cell-free DNA methylation analysis for the differential diagnosis of malignant pleural effusion and benign pleural effusion. This was a blind, prospective case–control biomarker study. We recruited 104 patients with pleural effusion for the study. We collected pleural fluid from patients with: MPE (n = 48), indeterminate pleural effusion in subjects with known malignancy or IPE (n = 28), and benign PE (n = 28), and performed the Sentinel-MPE liquid biopsy assay. The methylation level of Sentinel-MPE was markedly higher in the MPE samples compared to BPE control samples (*p* < 0.0001) and the same tendency was observed relative to IPE (*p* = 0.004). We also noted that the methylation signal was significantly higher in IPE relative to BPE (*p* < 0.001). We also assessed the diagnostic efficiency of the Sentinel-MPE test by performing receiver operating characteristic analysis (ROC). For the ROC analysis we combined the malignant and indeterminate pleural effusion groups (n = 76) and compared against the benign group (n = 28). The detection sensitivity and specificity of the Sentinel-MPE test was high (AUC = 0.912). The Sentinel-MPE appears to have better performance characteristics than cytology analysis. However, combining Sentinel-MPE with cytology analysis could be an even more effective approach for the diagnosis of MPE. The Sentinel-MPE test can discriminate between BPE and MPE. The Sentinel-MPE liquid biopsy test can detect aberrant DNA in several different tumor types. The Sentinel-MPE test can be a complementary tool to cytology in the diagnosis of MPE.

## Introduction

A malignant pleural effusion (MPE) forms when cells from either a lung cancer or another type of cancer spread to the pleural space. These cancer cells increase the production of pleural fluid and cause decreased absorption of the fluid. People with lung cancer, breast cancer, and lymphoma (a cancer of lymphoid tissue) are most likely to get an MPE. Mesothelioma (a rare cancer of the pleura itself) is another common cause of MPE. Other causes of MPE include cancers that have spread from the stomach, kidney, ovaries, and colon^1^.

The annual incidence of pleural effusion in the United States of America is estimated to be more than 1,500,000^2^. Lung cancer is the most common cause of malignant pleural effusion accounting for approximately 1/3 of MPE cases, followed by breast cancer, ovarian cancer, and gastrointestinal cancers; the primary tumor cannot be identified in 5–10% of MPE cases^3,4^. Nonmalignant, benign causes of pleural effusion (BPE) include congestive heart failure, tuberculous pleuritis, pneumonia, pulmonary embolism or infarction, cirrhosis, and collagen vascular disease^4^. Often patients with known malignancy will develop a pleural effusion and the cause (etiology) of the effusion remains indeterminate (IPE) after cytology examination^5^. Therefore, the etiology of pleural effusions has a wide differential diagnosis. A delayed diagnosis will directly affect subsequent treatment of patients and can be associated with markedly higher morbidity and mortality.

In standard care practice, cytology or pleural biopsy are typically used for diagnosing MPE^6^. The accurate and early detection of cancer cells in the pleural effusion is of significant clinical importance in the differential diagnosis of MPE. In 50% of lung cancer cases^7^ and 60% of all other types of cancers^8^, the malignant characteristics of a pleural effusion can be recognized by cytology. For cytologic diagnosis of malignancy, the return of positive cancer diagnosis is highest for adenocarcinoma and lowest for mesothelioma, squamous cell carcinoma, lymphoma, and sarcoma^4^. Repeated specimen collection with cytopathologic examination can yield an additional 27% increase in the malignancy rate^7^. However, there are limitations to cytopathology. For example, the sensitivity of cytological analysis depends on the volume of pleural fluid sampled, the number of specimens, the type of preparation and the experience of the examiner^9,10^. Furthermore, it is difficult to discern malignant from benign cells by morphology in the pleural fluid due to mesothelial and macrophage abnormalities. For instance, actively dividing mesothelial cells can mimic adenocarcinoma^11^. Pleural biopsy can accurately diagnose MPE, but this procedure is invasive procedure is not routinely used given the risk of complications^6^. Therefore, there is a need for new methods to diagnose malignancy in pleural fluid to prevent repeated diagnostic efforts and reduce harm to patients.

One means to improve the diagnosis of malignant pleural effusion could be by a liquid biopsy approach. A liquid biopsy involves examining cancer-related material (i.e., cell-free DNA, protein, exosomes) from blood or other body fluids. Liquid biopsy fluids contain cell-free DNA (cfDNA) in which the circulating tumor DNA (ctDNA) fraction may be present^12–16^; the ctDNA fraction varies based on tumor type and disease progression^17–19^. The presence of ctDNA fraction could be detected by testing cfDNA samples for tumor specific DNA methylation. DNA methylation biomarkers are more informative than DNA mutations since cancer specific DNA methylation occurs in a larger fraction of tumor samples than DNA mutations^20^. In addition, DNA methylation can be specific to multiple cancer types that develop in different organs and tissues^16^. Since cancers have many aberrantly methylated DNA regions^21–23^, multiple genomic loci can be investigated using DNA methylation-specific qPCR^24^ for the presence of tumor-specific DNA methylation and thus increase sensitivity of the test.

We have developed Sentinel-MPE, a novel robust liquid biopsy assay for cancer detection. The Sentinel-MPE assay is based on the detection of tumor specific DNA methylation at ten genomic loci^25^. Herein, we report on the diagnostic potential of the Sentinel-MPE test for the diagnosis of malignancy in PE.

## Results

### Characteristics of the patients

**Figure 1:**
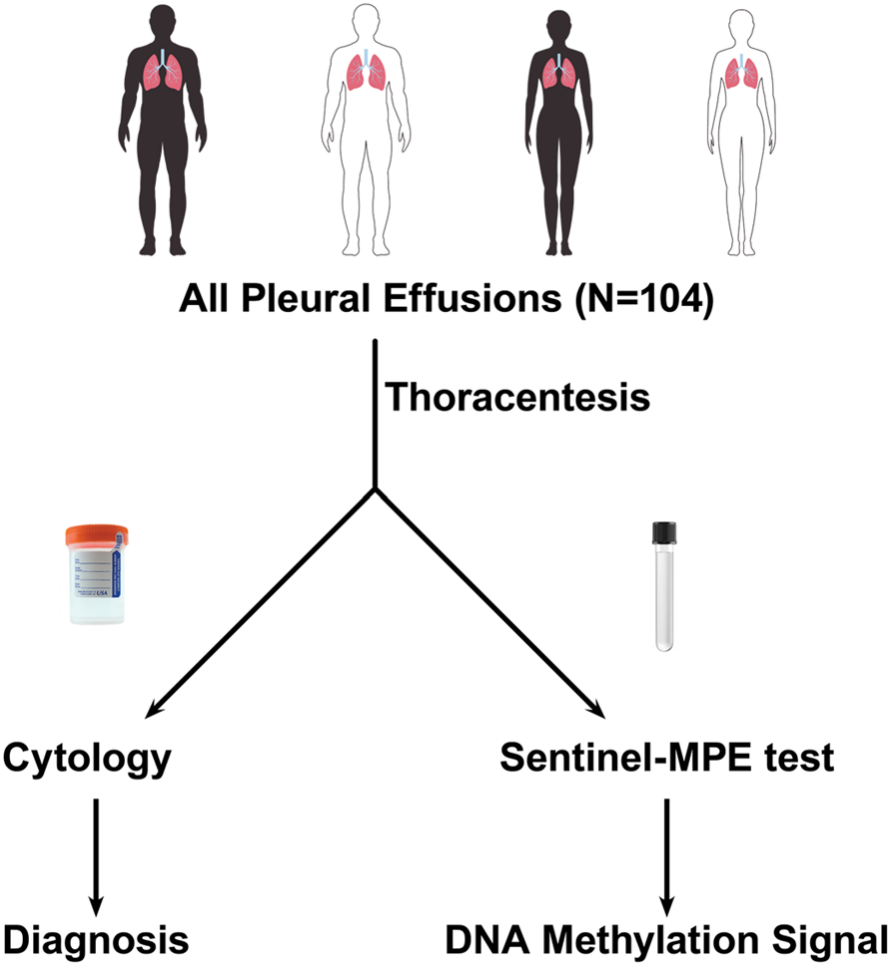
Study design for “all comers” pleural effusion for Sentinel-MPE liquid biopsy test. This was a prospective case control study with three different cohorts of pleural effusion disease benign pleural effusion subjects, paramalignant pleural effusion cases, and malignant pleural effusion individuals.

We prospectively collected pleural fluid from 104 patients undergoing diagnostic or therapeutic thoracentesis (Fig. 1). The study cohort included 45 males and 59 females, with a median age of 66.5 years (range 27–93). All demographic and clinical characteristics are summarized in Table 1. In the BPE group, there were 4 cases of chronic effusions from trapped lung. Three of the trapped lung cases were from uremia and the fourth case occurred after cardiac surgery.

**Table 1:** Demographic and Characteristics of Patients.

	Benign	Malignant	IPE
Age (years)			
Mean ± SD	62.8 ± 17.7	63.8 ± 14.4	68 ± 12.7
Range	29–93	27–83	27–92
Gender			
Male (45*, 43%)*	14 (50%)	20 (42%)	11 (39%)
Female (59*, 57%)*	14 (50%)	28 (58%)	17 (61%)
Tobacco use			
History (60*, 58%)*	19 (68%)	24 (50%)	17 (61%)
Never (44*, 42%)*	9 (32%)	24 (50%)	11 (39%)
Malignancy			
Lung		20	13
Breast		8	3
Hematologic		5	2
Ovarian		4	–
Uterine		2	2
Head/neck SCC		–	2
Hepatobiliary		1	1
GI NET		1	1
Sarcoma		2	–
Esophageal		-	1
Gastric		1	–
Germ cell		1	–
Thyroid		1	–
SFT high risk		–	1
EHE		–	1
Schwannoma		1	–
Mesothelioma*		1	–
Unknown**		1	–
BPE			
Infection	9		
Heart Failure	6		
Hemothorax	4		
Trapped lung	4		
Renal failure	3		
Cirrhosis	1		
VP shunt	1		

*MPE* malignant pleural effusion, *IPE* paramalignant effusion/indeterminate pleural effusion in a subject with known cancer, *SCC* squamous cell carcinoma, *GI NET* gastrointestinal neuroendocrine tumor, *SFT* solitary fibrous tumor, *EHE* epithelioid hemangioendothelioma, *BPE* benign pleural effusion, *VP* ventricular peritoneal.

*Peritoneal.

**Adenocarcinoma of unknown primary.

### Clinicopathological correlation analysis

We tested whether Sentinel-MPE DNA methylation levels were associated with the basic demographic characteristics of the patients. We did not find a significant association between Sentinel-MPE signal and patient age, gender, ethnicity or race (Figure 1A–D in the Supplement). There was a trend for African American subjects of higher methylation level (Figure 1D in the Supplement) than other racial groups. Finally, we did observe that the DNA methylation signal was significantly higher (*p* = 0.041) in exudative compared to transudative effusions (Figure 1E in the Supplement).

### The diagnostic performance of Sentinel-MPE liquid biopsy test for BPE, and all IPE, and MPE cancer types

We evaluated the diagnostic value of Sentinel-MPE using PE cfDNA specimens from 104 subjects. The Sentinel-MPE was positive with a number of common malignancies such as: NSCLC, breast, thyroid, gastric, esophageal, gall bladder, endometrial, and ovarian, clear cell and serous. Interestingly, it also detected MPE due to uterine cervical cancer, mesothelial cancer, plasma cell myeloma, acute myeloid leukemia (AML), chronic lymphocytic leukemia (CLL), thyroid cancer, and angiosarcoma. Whereas, the Sentinel-MPE test was negative in synovial sarcoma, malignant melanotic Schwannoma, thoracic seminoma, lung carcinoid, and epithelioid hemangioendothelioma. The later represent tumor types for which the assay was not specifically designed for.

**Figure 2:**
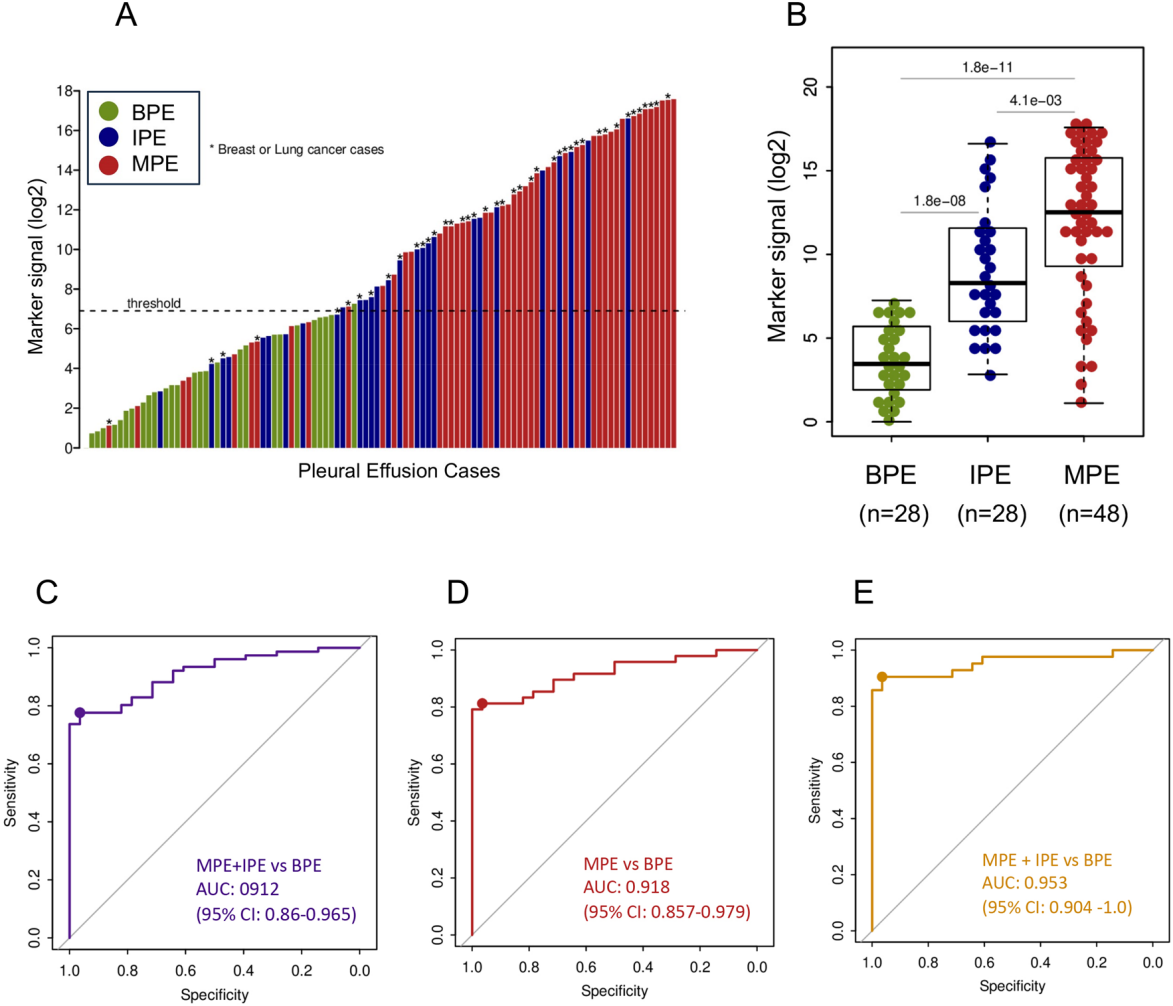
Performance of Sentinel-MPE liquid biopsy test in detecting malignancy in pleural effusion. (**A**) Waterfall plot displaying the methylation signal from the Sentinel-MPE liquid biopsy test for all 104 participants in the study. (**B**) Box plots displaying the mean methylation signal of benign pleural effusion (BPE), indeterminate pleural effusion (IPE), and malignant pleural effusion cohorts (MPE). (**C**) Receiver operating curve analysis of combined (MPE) and indeterminate effusion (IPE) groups compared to (BPE) group. The methylation signal levels were markedly higher in MPE and IPE groups relative to BPE group. (**D**) Receiver operating curve analysis of MPE versus BPE. There was an increase in the performance of the Sentinel-MPE liquid biopsy test. (**E**) Receiver operating curve analysis of combined MPE and IPE breast and lung cancer subjects compared to BPE subjects. Note the there was a further improvement in the performance of the Sentinel-MPE test for these two common cancer types.

The DNA methylation level of Sentinel-MPE was significantly increased in the MPE samples compared to BPE control samples (531-fold increased median, *p* < 0.0001) and a similar tendency was observed relative to IPE samples (19-fold increased median, *p* < 0.004) (Fig. 2A,B). The DNA methylation signal was also significantly higher in IPE relative to BPE (28-fold increased median, *p* < 0.0001), Fig. 2B). Next, we assessed the diagnostic performance of the Sentinel-MPE test by receiver operating characteristic analysis (ROC). First, we combined the MPE and IPE groups (n = 76) and compared them against the BPE group (n = 28). As shown in Fig. 2C, the performance of the Sentinel-MPE test was high (AUC = 0.912). Second, we compared the MPE group alone against the BPE group and found an increased performance of the liquid biopsy test (AUC = 0.918) (Fig. 2D).

### The diagnostic performance of Sentinel-MPE liquid biopsy test for benign and malignant disease arising from breast and lung cancer

Most malignant pleural effusions are secondary to metastatic involvement of the pleura from lung cancer or breast cancer^26^. In our cohort the majority of the MPE and IPE cases were subjects with lung and breast carcinomas (42/76, 55%). We performed additional analysis with breast and lung carcinoma MPE and IPE versus BPE. The DNA methylation signal was much higher in breast and lung carcinoma cases relative to control BPE cases (414-fold increased median, *p* < 2.1 × 10^–13^, Figure 2 in the Supplement) and there was an additional increase in the performance of the liquid biopsy test (AUC = 0.953, Fig. 2E).

### The diagnostic performance of the Sentinel-MPE liquid biopsy test compared to cytology

For all samples where data was available (n = 103, 75 malignant cases and 28 benign cases) we compared the diagnostic efficacy of the Sentinel-MPE test and traditional cytology to detect malignancy. We compared the ability of cytology alone, the Sentinel-MPE test, and both tests combined to detect malignancy in pleural effusion. Cytology analysis had a sensitivity of 49% (Table 2), whereas the Sentinel-MPE liquid biopsy test had a sensitivity of 76% (Table 2). There is further improvement in identifying malignant disease by combining both tests (sensitivity 84%, Table 2). This data indicates that the use of pleural effusion cfDNA methylation analysis using the Sentinel-MPE test could facilitate the early diagnosis of malignancy on the initial specimen collected.

**Table 2:** Diagnostic performance of Sentinel-MPE test for BPE and MPE.

Test	Accuracy (95% CI)	Sensitivity (95% CI)	Specificity (95% CI)	Positive predictive value (PPV) (95% CI)	Negative predictive value (NPV) (95% CI)
Cytology	0.631 (0.534–0.718)	0.493 (0.383–0.604)	1.000 (0.854–1.000)	1.000 (0.885–1.000)	0.424 (0.313–0.545)
Sentinel-MPE	0.816 (0.728–0.879)	0.760 (0.651–0.843)	0.964 (0.805–1.000)	0.983 (0.898–1.000)	0.600 (0.454–0.729)
Cytology + Sentinel-MPE	0.874 (0.794–0.926)	0.840 (0.739–0.907)	0.964 (0.805–1.000)	0.984 (0.907–1.000)	0.692 (0.534–0.814)

## Discussion

Malignant pleural effusion (MPE) is a frequent clinical problem in patients with neoplasia and represents advanced malignant disease with a poor prognosis with limited therapeutic options if not identified early. Patients with BPE can be managed with interventions that frequently provide resolution of the benign effusion or long-term control, with attention to the underlying benign etiology. Thus, it is essential to accurately distinguish between BPE and MPE for therapeutic decisions and to improve the prognosis of patients with MPE. Although there have been advancements in new imaging modalities, confirmation of malignant cells in the PE or pleural biopsy is necessary to establish a definitive diagnosis of MPE. Compared to pleural biopsy, cytological examination of pleural fluid represents a much less invasive procedure. However, cytology has limited diagnostic sensitivity (between 50 and 60%) due to substantial overlapping morphologic features among malignant, mesothelial, and reactive cells^27^. Pleural fluid is a source for liquid biopsy applying novel analyses to aid in the diagnosis of MPE. Here we report the development of molecular diagnostic assay to address these issues.

In agreement with previous studies^28,29^, we demonstrate that pleural fluid can be a sample source of liquid biopsy for the detection of malignancy. Lung cancer and metastatic breast cancer are two major causes of pleural effusion^26^. In the present study, we also showed that the Sentinel-MPE test could detect aberrant DNA methylation in both non-small cell lung cancer (NSCLC) and breast cancer cases with high sensitivity and specificity. An independent case control study of breast and lung cancer cases is currently underway.

Almost all cancers can potentially have pleural involvement causing MPE and many of them originate from cancer metastases^2,5^, making a test that can detect a variety of cancer type is deal. In the present study, we demonstrate that the Sentinel-MPE liquid biopsy test could detect malignancy in pleural effusions from several different cancer type such as: AML, colon cancer, lymphoma, and ovarian carcinomas.

Blood and sputum represent the two primary biofluid sources for liquid biopsy in the oncology literature. Pleural fluid, as a novel source, has significant advantages due to direct access to malignant cells and their microenvironment. Continued access to pleural fluid also allows characterization of targetable mutations for some patients^30^. Our study indicates that a molecular diagnostic test, based on DNA methylation analysis, could be of value in identification of MPE which could impact patient care.

Potential limitations to our study include the modest sample and studies with more patients are needed to validate the results in the future. To address this issue, we plan to conduct a larger multi-site clinical study to validate our initial results and give insight into the generalizability of the findings from the present study. Another limitation of the Sentinel-MPE test is that the assay did not detect most sarcomas or neuroendocrine tumors. Additional bioinformatics studies with genome wide methylation datasets are needed to identify DNA methylation markers for sarcomas and neuroendocrine cancers to include in the Sentinel-MPE test. We are currently conducting bioinformatic studies of sarcomas and neuroendocrine tumors to identify DNA methylation markers for these cancer types to add to the Sentinel-MPE test. Finally, whether the methylation changes of the Sentinel-MPE test correlate with the prognosis of MPE patients requires further study. In our planned larger validation study, we will explore the prognostic value of the assay.

The differentiation between MPE and indeterminate pleural effusion in patients with known malignancy (IPE) is important to ensure appropriate patient management^31^. Confirming MPE in patients with known cancer may upstage the cancer, thus leading to different therapeutic strategies such as curative versus palliative treatment in the present study, we noted a significant difference in the methylation signal between MPE and IPE. However, further studies are warranted to determine if such methylation signal differences can be exploited to discern MPE from IPE.

## Conclusions

The results from this study indicate that the Sentinel-MPE test displayed desirable performance characteristics in differentiating MPE from BPE. In addition, the Sentinel-MPE can detect aberrant DNA methylation in several different tumor types such as NSCLC, breast cancer, lymphoma, and acute myeloid leukemia, as well as thymic tumors, myeloma, angiosarcoma and mesothelioma. Importantly, the Sentinel-MPE can be a complementary tool for the cytopathologist to improve on the sensitivity and specificity of cytology alone in the diagnosis of MPE. Further research is required to assess the clinical utility, impact on patient outcomes, and cost-effectiveness of the Sentinel-MPE test.

## Methods

### Study design and participants

We performed a prospective case–control study (Fig. 1). We accrued pleural effusion samples from patients admitted to the hospital or seen in the pulmonology clinics at a single academic medical center The University of Arizona Human Subjects Protection Program approved the study and each participant provided written informed consent. Diagnostic cohort classifications were assigned by the study Principal Investigator as follows: Patients with no documented cancer were classified as having benign pleural effusion (BPE). Patients determined to have pleural involvement of cancer by cytology, biopsy, imaging, or clinical assessment were classified as having malignant pleural effusion (MPE). Patients with known malignancy and pleural effusion of unknown etiology with negative cytology were classified as indeterminate pleural effusion (IPE). Patient medical records were reviewed for pleural effusion cytology. Pleural effusion fluid samples were collected using Streck Cell-Free DNA BCT tubes (La Vista, NE) for DNA methylation analysis. The samples were assigned a study number and submitted to the laboratory in a blinded fashion. Cytology results were confirmed by two pathologists who were blinded to the Sentinel-MPE test results.

### DNA extraction and bisulfite treatment

Cell-free DNA was extracted from 2.0 ml samples of pleural effusion fluid as previously described by our group^25^. Cell-free DNA was isolated using the QIAamp Circulating Nucleic Acid Kit (Qiagen, Redwood City, CA, USA). The quantity of cfDNA was assessed by Qubit and Nanodrop instruments. Up to 500 ng of cfDNA was used for bisulfite treatment performed using EZ DNA Methylation-Gold Kit (Zymo Research, Irvine, CA, USA) as previously described^25^.

### DNA methylation analysis

The two-step methylation specific quantitative PCR was performed on QuantStudio 5 Real-Time PCR instrument using parameters we described before^25,32^. We have previously described the discovery of cancer specific DNA methylation genomic loci using TCGA Illumina HumanMethylation 450 microarray data^33^. We utilize the following methylation markers for Sentinel-MPE test: *MIR129-2, LINC01158, CCDC181, PRKCB, TBR1, ZNF781, MARCH11, VWC2, SLC9A3, and HOXA7.* The development of primer and probe sequences for 10 genomic loci have been previously described by our group^25^. In short, 10 qPCR amplicons specific for the marker loci and three control amplicons were designed. The marker amplicons were selected to overlap or be as close as possible to the marker CpGs determined by from the TCGA Illumina HumanMethylation450 microarray data^25,32^. In addition to 10 marker amplicons, 3 qPCR amplicons specific for universally methylated loci that serve as cfDNA load controls were designed. The pairs of primers and the probes for all qPCR amplicons were designed to be specific for the methylated sodium bisulfite treated DNA. The size of the amplicons was designed to be as short as possible (60–90 bp) to perform well on the fragmented cfDNA template. Primers and probes were designed to overlap at least 7 CpGs combined (at least two CpGs each, closer to the 3’ end for primers) to be specific only for the methylated template. The primers and the custom probes were manufactured by Integrated DNA Technologies (Coralville, IA, USA).

The cfDNA, isolated from 2 ml of plasma, was chemically modified with sodium bisulfite (BS) treated using EZ DNA Methylation-Gold Kit (Zymo Research, Irvine, CA, USA) according to the manufacturer’s instructions and eluted in 20 µl of water into low bind tubes. The first-round PCR amplification was executed in a 50 µl reaction volume using 25 µl of PerfeCta qPCR SuperMix Low ROX (Quanta Biosciences, Gaithersburg, MD, USA), 5 µl of 10 × mix of all amplicon primers (final concentration 385 nM each primer) and 20 µl of BS converted cfDNA. The reaction conditions were denaturation at 95 °C for 3 min, and then 15 cycles of 95 °C for 15 s, 57 °C for 30 s, and 72 °C for 30 s. The reaction product was then diluted 200-fold and used in the second-step qPCR. The qPCR mixture consisted of 10 µl of PerfeCta qPCR SuperMix Low ROX,500 nM each amplicon-specific primer, 200 nM amplicon-specific probe and 5 µl of the 200-fold diluted product from the first step in a 20 µl total reaction volume. The qPCR was conducted on QuantStudio™ 5 Real-Time PCR system (Thermo Fisher, Tempe, AZ), the reaction conditions were 95 °C denaturation for 3 min followed by 50 cycles of 95 °C for 15 s and 60 °C for 45 s.

### qPCR data analysis

We previously discussed the qPCR approach used for data analysis^25,32^. Briefly, the threshold cycles (Cts) for individual amplicons were determined using fixed marker-specific thresholds to keep consistency between individual qPCR runs. The Cts higher than 40 were set to 40. The data were then converted by a formula 40-Ct. This way Ct 40 was set as a background (zero) and the values that are still in log2 transformed scale but are increasing with the level of DNA methylation-specific signal were obtained. These values for all markers or the means of these values for all markers were used in the plots and ROC analysis.

### Statistical analysis

Statistical analyses were performed in R programming environment ver 4.3.1 (R Foundation for Statistical Computing, Vienna, Austria). Since the DNA methylation signal from the biomarkers spans several orders of magnitude, nonparametric tests were used to test differences between the groups (Wilcoxon rank sum test) or correlation between variables (Spearman's rank correlation coefficient). A two-sided *p* value < 0.05 was considered statistically significant. Receiver operating characteristic (ROC) analysis was performed using R library pROC to determine the areas under the curve (AUC) including 95% DeLong confidence intervals (CIs) and analytical threshold for the best accuracy (DNA methylation signal for the point at the ROC curve closest to the upper left corner (1.0,1.0)).The analytical threshold was used to classify samples as benign (DNA methylation signal bellow the threshold) or malignant (DNA methylation signal above the threshold) and to determine accuracy, sensitivity, specificity, positive predictive value (PPV) and negative predictive value (NPV) of the Sentinel-MPE assay (R library caret). For the combined assay of Sentinel-MPE and cytology, samples positive in either assay were considered positive, and samples negative in both assay classified as negative. The 95% Wilson score-based confidence intervals for the assay accuracy, sensitivity and specificity were determined.

All methods were performed in accordance with the relevant guidelines and regulations of the journal.

### Ethics approval

Institutional Review Board approval was obtained prior to the study initiation. All participants provided written informed consent.

### Supplementary Information

Supplementary Figures.

## Data Availability

All data generated or analyzed during this study are included in this published article and its supplementary information files.

## Abbreviations

AUC: Area under the curve
ROC: Receiver operating characteristic
cfDNA: Cell-free DNA
BPE: Benign pleural effusion
MPE: Malignant pleural effusion
IPE: Indeterminate pleural effusion
PPV: Positive predictive value
NPV: Negative predictive value
CI: Confidence intervals
Ct: Threshold cycle
