# Supplementary material for: Cell-free DNA methylation analysis as a marker of malignancy in pleural fluid
Source: Sci Rep. 2024 Feb 5;14:2939. doi: 10.1038/s41598-024-53132-x (PMC10844328; doi:10.1038/s41598-024-53132-x)
Supplement: Supplementary file 1 — Supplementary Figures. [file 41598_2024_53132_MOESM1_ESM.pdf]

Supplemental Figure 1

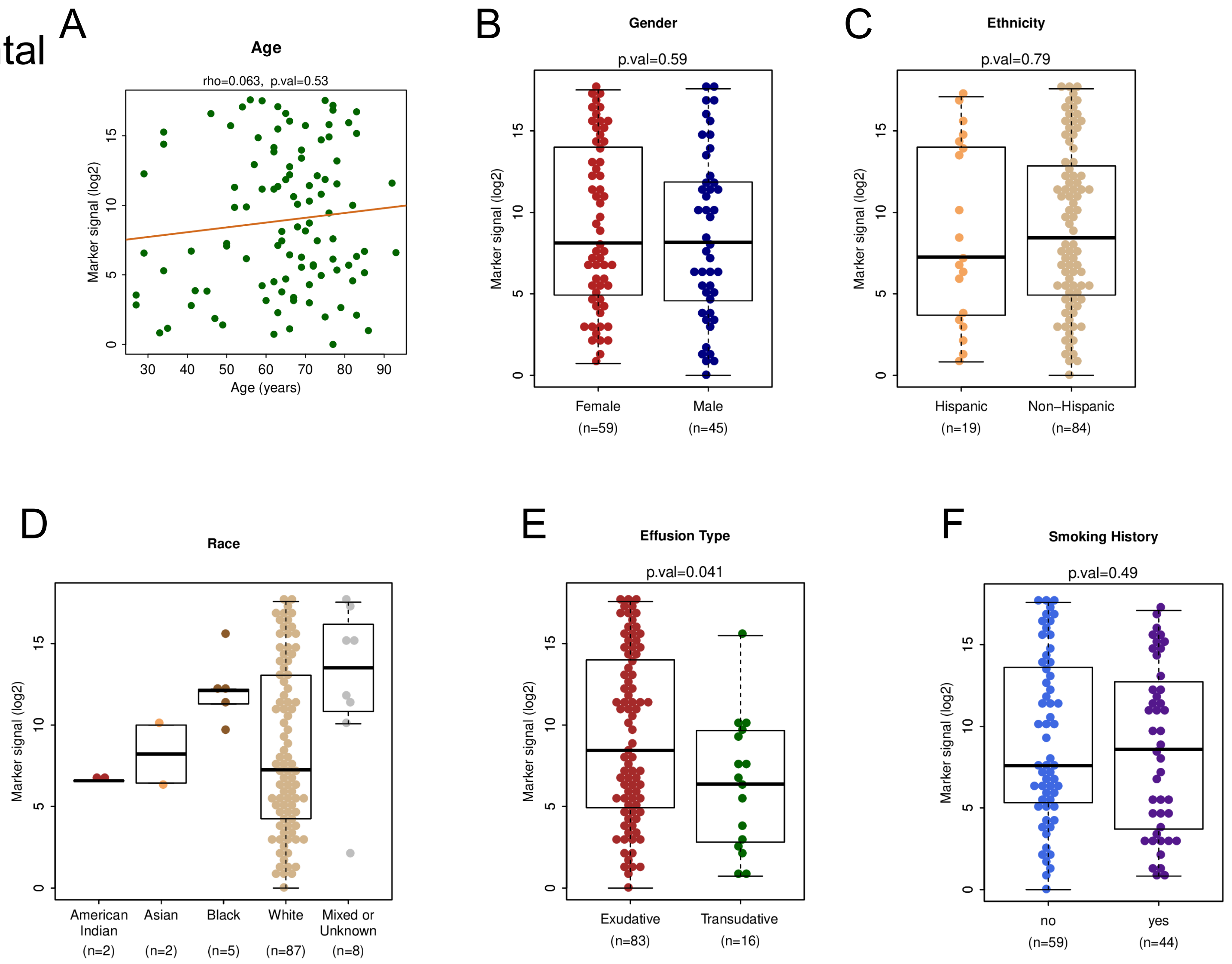

Suppmental Figure 2

...

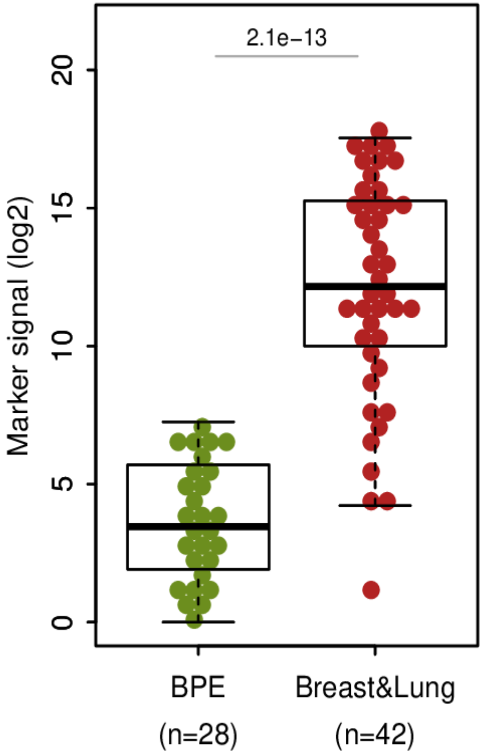

Supplemental Data.

Figure 1S. The analysis of the DNA methylation signal and its association with clinicopathologic features in pleural effusion samples. A) The testing of DNA methylation signal and age. B) The examination of DNA methylation signal and gender. C) The analysis of ethnicity and DNA methylation signal. D) The analysis of DNA methylation and race. E) The assessment of DNA methylation signal level in different types of pleural effusion. There was no significant correlation with age, gender, or race as Sentinel-MPE liquid biopsy methylation signal. However, there was a trend of black subjects to have a high methylation signal. There was a significant difference in the DNA methylation signal between exudative and transudative effusions.

Figure 2S. The Sentinel-MPE liquid biopsy test differentiates between MPE in breast and lung cancers and BPE controls with high sensitivity and specificity. The DNA methylation signal between breast and lung cancer cases versus benign disease cases. There was a markedly higher methylation signal in the MPE group compared to BPE.
